# Supplementary material for: Modeling Functional Motions of Biological Systems by Customized Natural Moves
Source: Biophys J. 2016 Aug 23;111(4):710–21. doi: 10.1016/j.bpj.2016.06.028 (PMC5002067; doi:10.1016/j.bpj.2016.06.028)
Supplement: Document S1. Supporting Materials and Methods and Figs. S1–S4 [file mmc1.pdf]

Biophysical Journal, Volume *111*

Supplemental Information

Modeling Functional Motions of Biological Systems by Customized Natural Moves

Samuel Demharter, Bernhard Knapp, Charlotte M. Deane, and Peter Minary

Modelling Functional Motions of Biological Systems by Customised Natural Moves - Supporting Material

S Demharter, Dr B Knapp, Prof CM Deane, Dr P Minary

Supporting Text

Natural move Monte Carlo sampling

The algorithm. Natural move Monte Carlo (NMMC) aims to sample the conformational space along user defined independent degrees of freedom X_i, which are described for both current models (for Case 1-2) in (1). Given this initial choice, the method generates canonical distributions along X_i over an effective energy surface $\tilde{E}$, which is defined by equation 1 below. Since the proposal kernel (1) along X_i is symmetrical, we use classical Metropolis Monte Carlo (2), which satisfies detailed balance, to sample the different states of X_i. Numerical experiments (3) demonstrate the accuracy (convergence to limiting distributions) and effectiveness (rate of convergence) of this approach.
In NMMC all degrees of freedom, X are partitioned into independent (X_i) and dependent (X_d) degrees of freedom (DoF). For example, X_i represent the independent orientational, translational or internal motions of structural fragments in a molecular chain, whereas X_d are the DoFs that are instantaneously minimised to facilitate exploration along X_i and preserve the integrity of the molecular chain(s) through chain closure(s). Thus, the effective potential over X_i is defined as

$$\tilde{E}(X_i) = \underset{X_d}{min}\Big\{E(X_i \cup X_d)\Big\}. \quad (1)$$

Therefore, natural move Monte Carlo is analogous to a Metropolis sampling (2) exploring state space spanned by X_i over the energy surface $\tilde{E}$. The most unique feature of NMMC is how the complex moves are generated. This is described below.

Implementation. The basic principle is that each new configuration during a proposal step is obtained via a combined chain breakage closure algorithm. This composite proposal kernel includes a stochastic proposal to update X_i followed by finding the most optimal (with respect to the new X_i) arrangement along X_d. This scheme can accelerate the conformational search for possible arrangements of *a priori* defined structural segments or regions (e.g. groups of segments) and is also free of any limitations caused by the lever-arm effects of distant torsional changes, which leads to increasingly (by chain length) low acceptance rates of dihedral moves. Thus, NMMC can be applied for any system regardless of size.
While the above description is general, the exact definition of independent X_i and dependent X_d degrees of freedom should be custom tailored to the model of interest. For the coarse-grained protein model of Case 1 and for the all-atom DNA model of Case 2 X_i and X_d are described in detail in (1).
Numerical experiments. In MCMC simulations it is generally regarded that an acceptance rate of ~0.4 is optimal when a single parameter (one independent variable of X_i) is updated and ~0.2 when a group of parameters (all independent variables in X_i) are updated. Given that we update X_i based on a multivariate normal distribution (1), we consider acceptance rates for natural moves in interval [0.2, 0.3] optimal and rates [0.15, 0.75] generally acceptable.
For replica exchange, we consider acceptance rates for adjacent temperature replica exchange of ~0.2 as optimal and rates in the interval [0.1, 0.3] as acceptable. The choice for these rates are based on considerations such as the sufficient relaxation time of individual Markov chains and the probability of 'coast to coast' visits of individual replicas.

Comparing results from different test cases

Each test case of the protocol is an independent model, in which the available conformational space is a subspace of C_f, the domain that includes all functionally relevant conformational variability. C_f is usually chosen to be a proper subspace of the

'full domain', C; $C_f \subset C$, e.g. C could be spanned by the Cartesian degrees of freedom (DoFs) and C_f by dihedral angles about single bonds and bond angles between bonds that an atom forms.

Each test case features some restricted set of DoFs spanning the state space $C_i \subseteq C_f \subset C$ for all $i = 1, , N_T$, where N_T is the number of test cases. Note, that the full domain, C is equipped with an energy function (the original energy surface), $E : C \to \mathbb{R}$ and the energy surface for a given test case is given by the function, E_i, which is a restriction of E to C_i and defined as $E_i : C_i \to \mathbb{R}, E_i(x) = E(x)$, for all $x \in C_i$. Thus, each test case is an independent model featured by C_i and the corresponding energy surface, E_i.

In spite of each test case being associated with its own state space, C_i distributions (over structural observables) obtained for different test cases can be compared to assess the contribution of a particular DoF (e.g. the relative motion of two adjacent helices enabled by a central kink) to functional motions (e.g. changes in MHC-II binding groove area and width). For example, let $\alpha : C \to \mathbb{R}$ be a structural observable and let $P_i(\alpha), i = 1, , N_T$ be the normalised numerical distributions over α we obtain for each test case via performing independent natural move Monte Carlo simulations covering each state space, $C_i, i = 1, , N_T$.

For the protein (Case 1) study we assess some features (e.g. bimodal) of these distributions $P_i(\alpha)$ to identify the DoFs that are essential and ones that are less critical to produce that feature, which may be linked to important biological function. For example, if the binding groove width distribution is bimodal then the MHC binding groove can exhibit two stable conformations (open and closed) even in the absence of the peptide. By systematically grouping all $P_i(\alpha)$ that exhibit this behaviour from those that do not, we can identify the underpinning essential DoFs responsible for this phenomenon. In a similarly qualitative but systematic approach the DNA (Case 2) study compares distributions for test cases to purely identify the existence and directionality of effects a chemical modification imposes on the DNA structural parameters. Our robust initial search can identify test cases or phenomena that could be further investigated by molecular dynamics to obtain refined quantitative information.

Supporting Figures

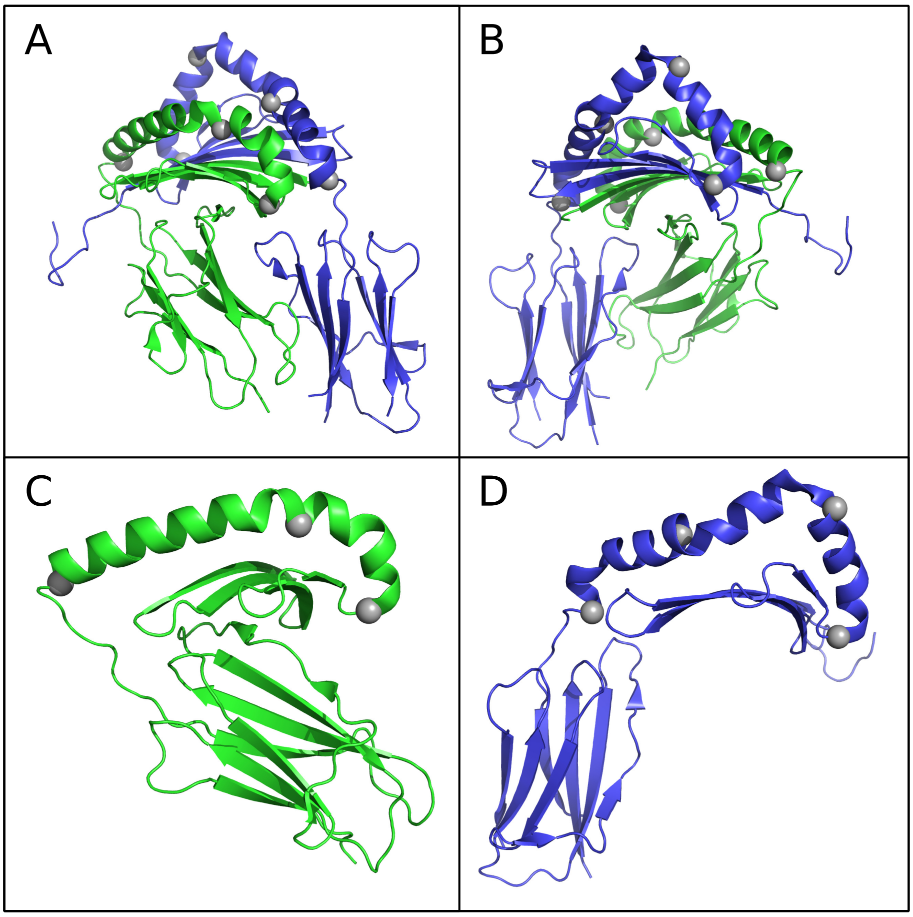

Figure S1: **Molten zones in MHC II. A,B** MHC II is shown in cartoon representation. Chains A and B are coloured in green and blue, respectively. The molten zones are depicted as grey spheres. **C** Chain A and its three molten zones are shown. **D** Chain B and its four molten zones are shown.

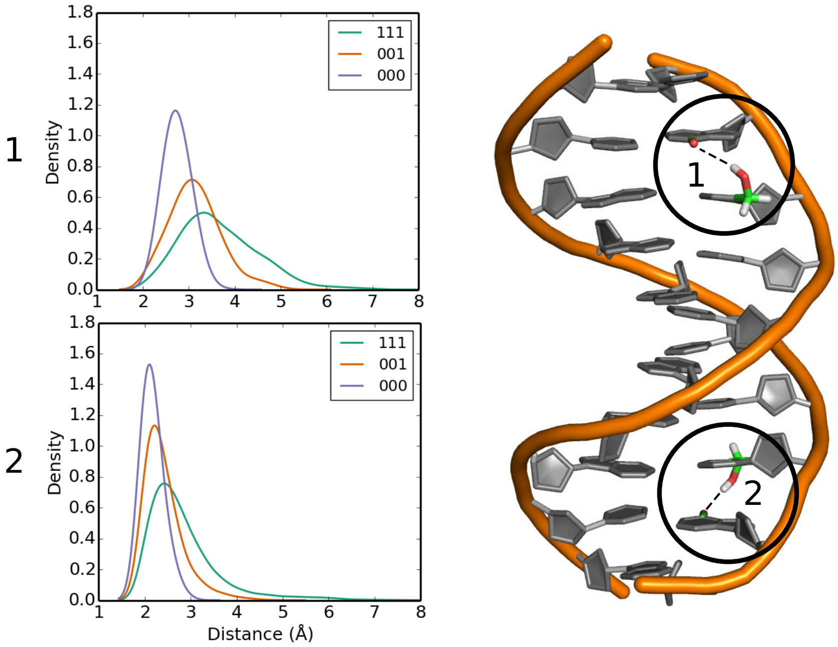

Figure S2: **The effect of customised natural moves on an intra-strand hydrogen bond.** Distance distributions of the two non-canonical hydrogen bonds between the hydroxyl hydrogens on 5hmC and the O6 oxygen of the 3'-adjacent guanine as highlighted on the right. All three test cases are shown. The X-ray structure, which we used as our starting structure, is not totally symmetric so we do not expect totally symmetrical effects as we move from ${}^{111}T$ to ${}^{000}T$.

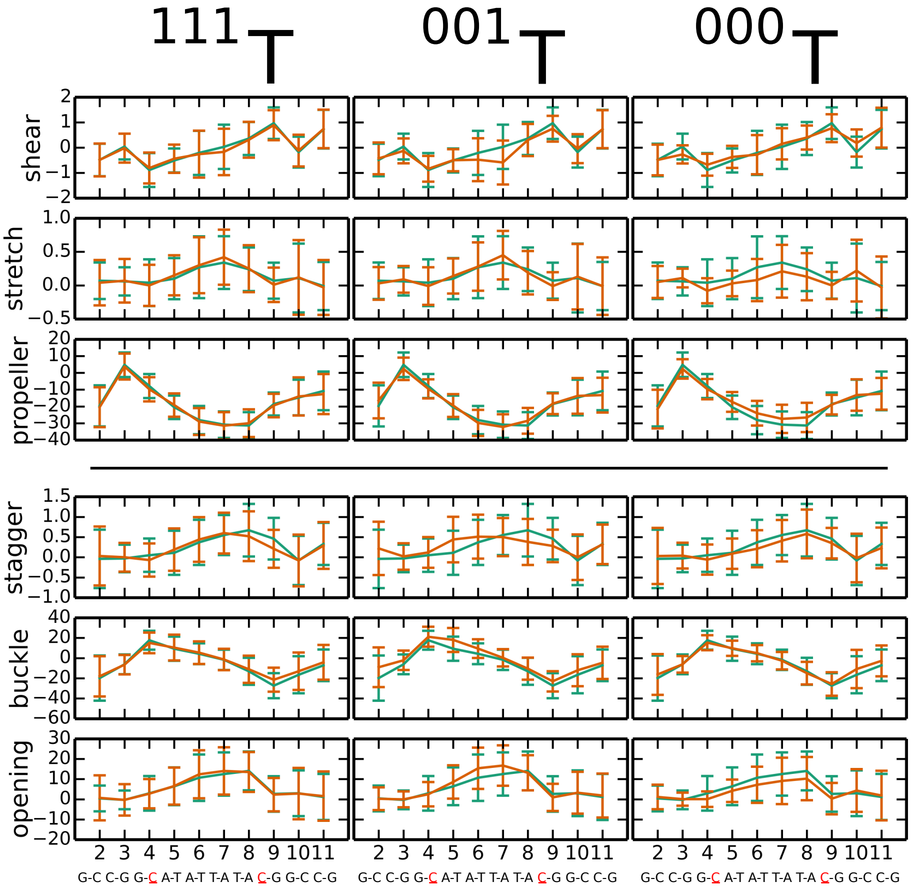

Figure S3: **The effect of three test cases on the base pair parameters.** The parameters for all base pairs in the three test cases ^{111}T, ^{001}T and ^{000}T (orange) are compared against the control simulation without modification (green). The top half of the figure shows the three parameters shown in Fig 3. All other parameters do not show any systematic changes caused by the customised natural moves. Displacement parameters (shear, stretch, stagger) are shown in Ångstrom and angular parameters (buckle, propeller, opening) are shown in degrees. The vertical bars show the standard deviation. The red underlined characters show the positions of the epigenetic mark.

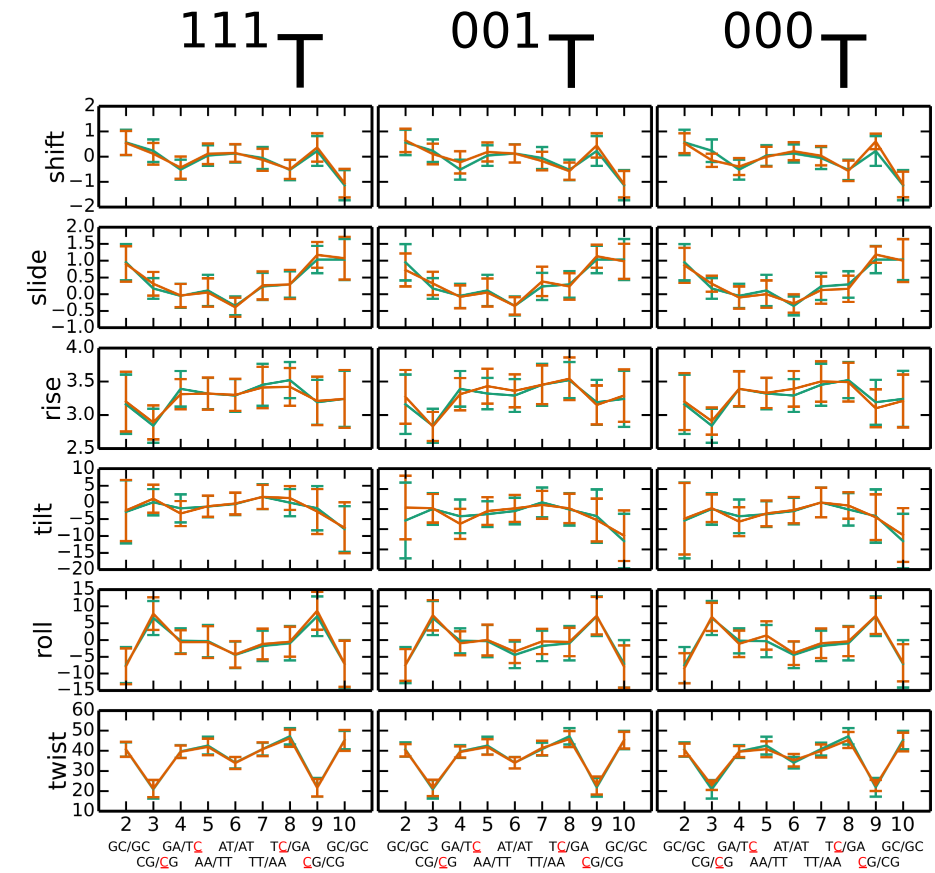

Figure S4: **The effect of three test cases on the base stack parameters.** The parameters for the three test cases ^{111}T, ^{001}T and ^{000}T (orange) are compared against the control simulation without modification (green). No systematic changes were observed in any of the test cases. Displacement parameters (shift, slide, rise) are shown in Ångstrom and angular parameters (tilt, roll, twist) are shown in degrees. The vertical bars show the standard deviation. The red underlined characters show the positions of the epigenetic mark.

Supporting References

1. Minary, P., and M. Levitt, 2010. Conformational optimization with natural degrees of freedom: a novel stochastic chain closure algorithm. J. Comput. Biol. 17:993–1010.
2. Metropolis, N., A. W. Rosenbluth, M. N. Rosenbluth, A. H. Teller, and E. Teller, 1953. Equation of State Calculations by Fast Computing Machines. J. Chem. Phys. 21:1087.
3. Sim, A. Y. L., M. Levitt, and P. Minary, 2012. Modeling and design by hierarchical natural moves. P. Natl. Acad. Sci. 109:2890–5.
