# Supplementary material for: Modeling Functional Motions of Biological Systems by Customized Natural Moves
Source: Biophys J. 2016 Aug 23;111(4):710–21. doi: 10.1016/j.bpj.2016.06.028 (PMC5002067; doi:10.1016/j.bpj.2016.06.028)
Supplement: Document S2. Article plus Supporting Material [file mmc2.pdf]

Biophysical Journal
Article

Modeling Functional Motions of Biological Systems by Customized Natural Moves

Samuel Demharter,[1] Bernhard Knapp,[2] Charlotte M. Deane,[2] and Peter Minary[1,*]
[1]Department of Computer Science and [2]Department of Statistics, University of Oxford, Oxford, UK

ABSTRACT Simulating the functional motions of biomolecular systems requires large computational resources. We introduce a computationally inexpensive protocol for the systematic testing of hypotheses regarding the dynamic behavior of proteins and nucleic acids. The protocol is based on natural move Monte Carlo, a highly efficient conformational sampling method with built-in customization capabilities that allows researchers to design and perform a large number of simulations to investigate functional motions in biological systems. We demonstrate the use of this protocol on both a protein and a DNA case study. Firstly, we investigate the plasticity of a class II major histocompatibility complex in the absence of a bound peptide. Secondly, we study the effects of the epigenetic mark 5-hydroxymethyl on cytosine on the structure of the Dickerson-Drew dodecamer. We show how our customized natural moves protocol can be used to investigate causal relationships of functional motions in biological systems.

INTRODUCTION

Functional motions in biomolecules are central to many biological processes (1). Molecular simulations are often used as a tool to investigate these dynamics and interpret (2,3) and/or refine (4) experimental data or inspire new experiments (5).

Large improvements in computational resources and algorithms have been made since the first molecular simulation of a protein in 1977 (6,7). Recent milestones include the 50-ns molecular dynamics (MD) simulation of the full satellite tobacco mosaic virus with 1,000,000 particles (8), the Folding@Home project that used >400,000 personal computers to study challenging problems such as protein folding (9), and a study that presented millisecond simulations to study the folding pathways of small fast-folding proteins (10).

Despite these advances, the high dimensionality and complex energy surfaces still pose a challenge for simulations of large biomolecules (11,12). In an effort to address these limitations there have been promising developments in dimensionality reducing methods that exploit the inherent modularity and collective motions in biomolecules (13,14). For example, essential dynamics coarse-graining (ED-CG) identifies sites that reflect the essential dynamics of an atomistic molecular dynamics trajectory (15). Other methods based on elastic network models, principal component analysis, and normal mode analysis have also been successfully used to study functional motions in biomolecules (16–18). While these methods are not as physically accurate as MD simulations, their increased sampling efficiency makes them a valuable tool to generate new hypotheses that can be tested by experiments. One of the main challenges of these methods, however, is finding a set of degrees of freedom (DOFs) that describe the system accurately enough to draw biologically relevant conclusions (14). Thus, it is of value to have computationally cheap methods that allow for the easy manipulation of DOFs to test different hypotheses about the functional motions of biomolecules in silico.

One method designed to address the dimensionality challenge is natural-move Monte Carlo (NMMC) (19,20). NMMC is a conformational sampling method that exploits the modular nature of biomolecules to accelerate the exploration of structural landscapes to identify functionally relevant conformations. Instead of sampling the position of each atom in the system, groups of atoms or residues that are part of a shared structural region can be grouped and moved collectively. This gives rise to a conformational sampling strategy that considers the system as a collection of structural regions and exclusively samples their arrangements along the user-defined DOFs. Thus, this method reduces dimensionality by several orders of magnitude by sampling

Submitted February 1, 2016, and accepted for publication June 22, 2016.
*Correspondence: peter.minary@cs.ox.ac.uk
Editor: Tamar Schlick.
http://dx.doi.org/10.1016/j.bpj.2016.06.028
© 2016 Biophysical Society.
This is an open access article under the CC BY license (http://creativecommons.org/licenses/by/4.0/).

along generalized coordinates. While it does not directly reveal any kinetic information, it can rapidly generate ensembles of thermodynamically feasible structures that appear according to canonical probabilities using computational resources that are readily accessible. In a recent study we showed that NMMC yields comparable results to and is three orders-of-magnitude faster than conventional MD when simulating peptide detachment from class I major histocompatibility complex (MHC I) molecules (21).

Traditionally NMMC is used to explore the conformational landscape along a particular set of DOFs chosen by the researcher. Several studies of DNA and protein systems have followed this approach (19–25). However, the initial choice of DOFs might not always be optimal. Additionally, if the objective is to investigate the causality of functional motions, it may be informative to perform NMMC simulations for a variety of sets of DOFs.

Here we introduce a protocol based on customized natural moves (cNMs) to address the challenge of choosing suitable DOFs and to allow for the systematic investigation of hypotheses regarding functional motions in biomolecules. We use cNMs to modulate translations and rotations of segments as well as torsion and bend angles of bonds and compare different sets of cNMMC simulations to infer causal relationships in functional motions. We use two case studies to demonstrate its application. In the first, we investigate functional motions in the class II major histocompatibility complex (MHC II) and in the second, we study the structural effects of an epigenetic mark on a DNA model system.

The MHC II is a transmembrane protein that presents potentially harmful peptides to CD4+ T-cells (26). The structure of the peptide-loaded MHC II binding groove is well documented (27); however, to date no structure has been solved for the peptide-free MHC II (28) due to its dynamic nature. Several studies suggest that the absence of peptide destabilizes the MHC II structure (29,30). Using our protocol, we investigated the functional motions involved in the destabilization of the peptide-free MHC II complex.

We designed multiple sets of cNMs and performed NMMC simulations to study the plasticity of the empty MHC II binding groove. Our simulations suggest that the $\beta 1$ helix can assume a number of transitory states that cause a narrowing of the binding groove in the absence of peptide.

We also demonstrate our protocol on the structure of the Dickerson-Drew dodecamer (DDD) that was modified with the 5-hydroxymethylcytosine (5hmC) epigenetic mark. 5hmC is generated by the catalytic activity of oxygenases on 5-methylcytosine (5mC). 5mC is known to increase dsDNA stability, which is consistent with its role in gene expression at CpG islands (31). Given the right sequence context, 5hmC can partly reverse the stabilizing effect of 5mC (32). Lercher et al. (33) have observed two configurations of this epigenetic mark on the DDD, one of which formed a hydrogen bond with the $3'$-adjacent guanine. We hypothesized that this noncanonical hydrogen bond interferes with the canonical hydrogen-bonding of the neighboring basepair. To test this hypothesis, we defined a set of cNMs designed to amplify the structural effect of the epigenetic mark on the $3'$-adjacent canonical basepair.

Due to an increase of several orders of magnitude in computational efficiency compared to atomistic Cartesian sampling methods and its inherent capability to customize DOFs, the cNMMC protocol can facilitate multiple simulations of large biological assemblies. This allows for a side-by-side comparison of different sets of cNMs and enables the testing of hypotheses regarding functional motions in biological systems.

MATERIALS AND METHODS

NMMC

A molecular system can be defined as a collection of monomers or more formally a set, $\Omega = \{m_1, \ldots, m_N\}$, where m_i for $i = 1, \ldots, N$ refers to the residues of a structure. Sequentially numbered residues can be grouped into chains, $\mathbf{C} = \{C_1, \ldots, C_{N_c}\}$, where $C_i = \{m_{c_h^i}, m_{c_h^i+1} \ldots, m_{c_t^i}\}$ (e.g., c_h^i and c_t^i are indices of the head and tail residues of chain i) for $i = 1, \ldots, N_c$. The chain concept can be further generalized into a group of segments, $\mathbf{S} = \{S_1, \ldots, S_{N_s}\}$, where $S_i = \{m_{s_h^i}, m_{s_h^i+1} \ldots, m_{s_t^i}\}$ for $i = 1, \ldots, N_s$ (e.g., s_h^i and s_t^i are indices of the head and tail residues of segment i).

After defining the segments, we can introduce the set of residues that make up the segments:

$$\Omega_s = \bigcup_{i=1}^{Ns} S_i \,\Big|\, \Omega_s \subseteq \Omega. \tag{1}$$

At each iteration in the simulation, the segments are moved in a Monte Carlo fashion along user-defined DOFs, which collectively are called natural moves; these may include translations and rotations of segments as well as torsion and bend angles within segments. After each propagation step, the set of atoms (or entire residues) connecting two segments are rearranged by a linear complexity chain closure algorithm (19). We will refer to this set as the molten zone (MZ). This allows for the reconstruction of chain breaks that may result from the movement of the segments.

Thus, the residues outside of the segments form the set of MZ residues

$$\Omega_m = \Omega \setminus \Omega_s = \bigcup_{k=1}^{N_{MZ}} \Omega_{MZ}^{(k)}, \tag{2}$$

where N_{MZ} is the number of MZs and $\Omega_{MZ}^{(k)}$ is the set of residues in the kth molten zone.

Furthermore, we define $\Omega_{MZ} = \{\Omega_{MZ}^{(1)}, \ldots, \Omega_{MZ}^{(k)}\}$ as the set of molten zones.

In this study, $\Omega_s \subset \Omega$ (Ω_s is a proper subset of Ω), thus $\Omega_m \neq \emptyset$ for proteins. For nucleic acids we define each residue as a segment, therefore $\Omega_m = \emptyset$. In this case we use $*\Omega_{MZ}$ to denote the set of molten zones $*\Omega_{MZ}^{(j)}, j = 1, \ldots, k$, where $*\Omega_{MZ}^{(j)}$ refers to a molten zone with a set of atoms that are used by the closure algorithm to connect chain breaks that may be caused by moving adjacent nucleotides independently.

cNMs

cNMs are natural moves that can be modified to investigate functional motions in biomolecules. cNMs include translations and rotations of segments, which may also exhibit internal flexibility such as torsion and bend angles

of bonds. cNMs can be created by grouping two segments into one so that they move as a unified segment. Instead of two independent segments that are moved separately, there is now a single set of natural moves that describes the collective motion of both segments. This can be useful, for example, to test whether flexibility in an α-helix kink is important for a particular functional motion or to explore how different levels of collective motion may affect a structural mechanism. Customization may in addition occur at the level of internal flexibility of segments. When internal flexibility is disabled, segments are treated as rigid bodies. When internal flexibility is activated, torsion angles around bonds are changed along with the movement of segments. cNMs also allow us to selectively activate or deactivate sampling of torsional rotations around specific bonds.

The protocol

We devised a protocol based on cNMs to investigate functional motions in biomolecular structures. The key steps of the protocol: Step I—define a hypothesis; Step II—translate hypothesis into natural moves; Step III—activate/inactivate natural moves to generate test cases for the investigation of the hypothesis; and Step IV—perform conformational sampling on each test case and evaluate the results with respect to the hypothesis. The steps are described in more detail below.

Step I: define a hypothesis

This step, which is often left to the user, is based on observing experimental data in the literature and/or biological intuition about functionally relevant flexibility, the rigidity or collective motion of atoms, and secondary or even tertiary structure elements. While this step cannot be entirely generalized, it facilitates the engagement of applied scientists, who often have extensive knowledge of the system of interest, in the design of computational experiments so that their hypothesis can be tested.

Step II: translate hypothesis into natural moves

Based on this hypothesis, an initial set of natural moves can be defined that encapsulates all movements that the researcher specifies as important for the functional motion. These might be residues and/or larger segments as well as the torsion and bend angles of individual bonds.

Step III: generate test cases

After the initial set of natural moves has been defined, it is then possible to generate different sets of cNMs by selectively deactivating certain DOFs to study their effect on functional motions. Natural moves may be customized by modulating the DOFs that describe the movement of segments (translations and rotations) as well as their internal flexibility (torsion and bend angles of bonds).

The relative movement of two segments may result in a chain-break that is closed by the rearrangement of atoms in the molten zone. When two segments are grouped into a bigger segment, the relative orientation of these segments to each other is maintained throughout the simulation.

Here, we consider a molten zone $\Omega_{MZ}^{(k)}$, for $k = 1, \dots, N_{MZ}$ to be active when the segments on either sides are moved independently and inactive otherwise. For clarity, we only present the case where MZs are made up of residues. For molten zones made up of atoms, we use $^*\Omega$ instead of Ω.

Formally we can introduce a function $f: \Omega_{MZ} \rightarrow \{0,1\}, f(\Omega_{MZ}^{(k)}) = 1$ if $\Omega_{MZ}^{(k)}$ is enabled, which leaves corresponding S_j and S_{j+1} as independent segments and $f(\Omega_{MZ}^{(k)}) = 0$ if $\Omega_{MZ}^{(k)}$ is disabled, which fuses the two adjacent segments into one, e.g., $S_{(j,j+1)}$.

Similarly, some segments may have internal flexibility such as bond torsion angles. Therefore, it is possible to introduce a set of torsion angles $\Omega_\phi^{(l)}$, for $l = 1, \dots, N_\phi$ and define a function $g: \Omega_\phi \rightarrow \{0,1\}$, where $g(\Omega_\phi^{(l)}) = 1$ if $\Omega_\phi^{(l)}$ is active and $g(\Omega_\phi^{(l)}) = 0$ if $\Omega_\phi^{(l)}$ is inactive.

The decomposition and the internal flexibility of segments in a structure may be represented by a vector $\mathbb{D}$ in which each element refers to the state of a specific MZ or torsion angle. $\mathbb{D}$ may be defined as:

$$\mathbb{D} = \left\{ f\left(\Omega_{MZ}^{(1)}\right), \dots, f\left(\Omega_{MZ}^{(N_{MZ})}\right), g\left(\Omega_\phi^{(1)}\right), \dots, g\left(\Omega_\phi^{(N_\phi)}\right) \right\}, \tag{3}$$

where Ω_{MZ} and Ω_ϕ refer to the respective molten zones and torsion angles and $|\mathbb{D}| = N_{MZ} + N_\phi$.

Thus, given two functions, f and g, leads to a decomposition $\mathbb{D}(f,g)$, which we associate with a test case $^{\mathbb{D}(f,g)}T$ (see Eq. 3 for the definition of $\mathbb{D}$).

Test cases are associated with a set of functions, e.g., $f_i, i = 1, \dots, N_T$ where N_T is the number of test cases. If we have three molten zones, then there are $2^3 = 8$ different test cases that result from eight functions with identical domains $\{\Omega_{MZ}^{(1)}, \Omega_{MZ}^{(2)}, \Omega_{MZ}^{(3)}\}$ and codomains $\{0,1\}$, but unique functional maps.

A particular test case may allow for flexibility in an α-helix kink while another test case treats the helix as rigid. Similarly, a selected torsional rotation around a bond may be sampled freely in one test case, while in another test case the dihedral angle is maintained throughout the simulation. This capability allows the researcher to investigate causal relationships between structural features and biophysical mechanisms.

Step IV: conformational sampling and evaluation

Each test case implies a unique set of DOFs (cNMs) that can be sampled with NMMC. The resulting distributions can then be evaluated with respect to the initial hypothesis. Below we outline the method details for both of our case studies. Note that for reproducible results, replica simulations are needed (21,28,34).

Implementation details

Natural moves change the orientation, position, and internal state (dihedral and bond angles) of structural segments, which are connected by the remaining atoms (e.g., coarse-grained or actual) in the system. These remaining atoms bridge segments and constitute the molten zones (19).

While Minary and Levitt (19) describes the anatomy of structural segments and MZs for both models (Cases 1 and 2) in detail, this article aims to provide a high-level practical annotation of natural moves using binary strings. (The implementation details on how to convert these binary strings into customized natural moves is described in "A Tutorial for the Customized Natural Move protocol", which is available at http://www.cs.ox.ac.uk/mosaics/examples/functional_motions_cNMMC.html.)

Simulation details

All simulations were carried out with the MOSAICS software package (35). All distributions were plotted with matplotlib (36) and pandas (37) using a bandwidth of 0.1.

Protein (MHC II)

NMMC simulations were initiated from an x-ray structure of the MHC II (HLA-DR) in complex with HLA-DM at a resolution of 2.6 Å (PDB: 4GBX) (38). The structure was coarse-grained using a 3-point-per-residue protein model (22). We generated the MHC II model by removing the HLA-DM part of the structure file. To ensure extensive conformational sampling, we performed parallel tempering using six replicas at temperatures 300, 336, 376, 421, 472, and 529 K. We ran 15 independent repeats for each test case. Each repeat was run for 1,000,000 Monte Carlo iterations. These parameters were chosen so that the acceptance rates within each replica and the interreplica exchange rates were at least 0.25 and 0.1, respectively. All data were collected from the replica with a canonical temperature of 300 K. Distances were calculated with MDAnalysis (39) and the binding-groove

surface area was calculated using differential geometric analysis as described in Hischenhuber et al. (40).

DNA (Dickerson-Drew dodecamer)

The Dickerson-Drew dodecamer (DDD) in configuration A (5-hydroxymethyl epigenetic marks point toward the O6 oxygen of the 3′-adjacent guanine (G/O6)) (33) at 1.3 Å resolution was used as the starting point. The missing hydroxyl hydrogens were added and oriented toward the 3′-adjacent G/O6. Hydrogens were added to the remaining atoms using pymol1.7's h_add command (41). The 3′- and 5′-terminal basepairs were removed. An all-atom representation was used with the Amber99-bsc0 force field (42) and a dielectric dampening model (43). Using this model, we reproduced/predicted experimental nucleosome occupancy up to a resolution of a few nucleotides (44). Single-temperature natural-move Monte Carlo was performed at 300 K. We ran 30 independent repeats of 5,000,000 Monte Carlo iterations for each test case. Helical parameters were analyzed using x3DNA (45).

RESULTS

Here we demonstrate a protocol, based on customized natural moves, which allows the user to design and perform multiple simulation test cases to investigate the causal relationship between different structural features and functional motions. The protocol is computationally cheap and has in-built customization capabilities, which makes the design and run-time of large numbers of customized simulations easily accessible for most research groups.

Initially a hypothesis is defined regarding the functional motions of a particular biomolecule. The hypothesis is used to design natural moves that can be systematically turned on/off to test their involvement in a particular functional motion. In the following two case studies, we show how cNMs may help to understand the causality underlying functional motions in biomolecules.

Case 1: the plasticity of the empty MHC II binding groove

MHC IIs are transmembrane proteins expressed by antigen-presenting cells that are critical for the activation of the adaptive immune response in vertebrates (46,47). Peptides derived from extracellular proteins that bind the MHC II binding groove inside the cell are transported to the surface and are recognized by receptors on the surface of CD^{4+} helper T-cells (48). While several MHC II crystal structures with high structural similarity have been solved in the presence of peptide (49), the MHC II structure devoid of peptide has not been solved to date (28).

In the absence of peptide, the MHC class II binding groove can take on kinetically distinct forms that are either receptive or averse to peptide binding (29). The receptive state mainly exists straight after peptide dissociation and has a half-life of a few minutes after which the MHC II takes on a peptide averse state (30,50–52). Structural changes in the binding groove have been implicated in this process (53,54). In this case study we demonstrate how cNMs may be used to investigate the plasticity of the empty MHC II binding groove. Here we follow the general steps introduced in the Materials and Methods.

Step I: define a hypothesis

The literature suggests there are several structural features that may contribute to the plasticity of the empty binding groove. The C-terminal region of helix $\alpha 1$ has been shown to exhibit a distinct conformation in the absence of peptide by mass spectrometric mapping (54). This region is also part of the binding site for the peptide-loading chaperone HLA-DM, and undergoes a structural change upon binding HLA-DM (38). Therefore, we included this structural feature as an area of potential flexibility by introducing a molten zone at the C-terminal end of the $\alpha 1$ helix ($\alpha 1$-1 in Fig. 1 *A*).

Residues $\beta 53$–68 on helix $\beta 1$ are part of epitopes for conformation-sensitive antibodies that are selective for the empty binding groove (53,55). This region has been shown to undergo local structural changes by circular-dichroism spectroscopy (53). MD simulations and comparison of experimental MHC II structures revealed structural variability around a sharp kink in this region (49,56,57). Given these observations, we introduced a further MZ at the N-terminal kink of the $\beta 1$ helix ($\beta 1$-1 in Fig. 1 *A*).

The second kink on the $\beta 1$ helix has not been implicated in major structural changes. This is likely due to a disulphide bridge anchoring a conserved cysteine to the β-floor below. However, the segments on either side might still be influenced by flexibility in this kink, so a third molten zone was introduced at this point ($\beta 1$-2 in Fig. 1 *A*).

Thus, our hypothesis states that conformational flexibility in the three unstructured regions in the two helices ($\alpha 1$ and $\beta 1$) contributes to the variability of binding groove width and area in the empty MHC II complex.

Step II: translate hypothesis into natural moves

Our hypothesis on binding groove flexibility provided us with a starting point for defining an initial set of segments, which can undergo three body rotations and translations. This resulted in an initial decomposition consisting of five segments (Fig. 1 *B*). We used secondary structure information to place MZs between these segments. In this coarse-grained protein case study we did not include any internal flexibility within the segments.

Step III: generate test cases

In this simple scenario, each of the three MZs may either be enabled or kept rigid, thereby splitting or grouping two neighboring segments. As a result, there are $2^3 = 8$ different possible test cases that may be generated. For example, test case ${}^{010}T$ refers to a system in which MZs $\Omega_{MZ}^{(1)}$ and $\Omega_{MZ}^{(3)}$ are deactivated and $\Omega_{MZ}^{(2)}$ is activated. This creates three regions (one in helix A and two in helix B), as shown in Fig. 1 *C*. Table 1 presents the remaining test cases.

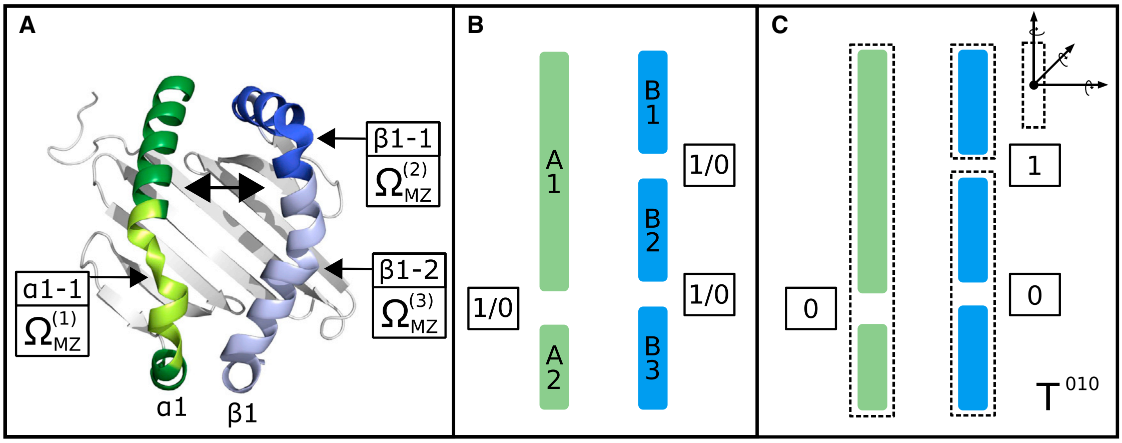

FIGURE 1 Decomposing the MHC II binding groove into natural moves. (*A*) Cartoon representation of the MHC II binding groove (peptide not shown). The three positions α1-1, β1-1, and β1-2 where we defined molten zones $\Omega_{MZ}^{(1)}$, $\Omega_{MZ}^{(2)}$, and $\Omega_{MZ}^{(3)}$ are highlighted by arrows. Helix α1 is shown in green; helix β1 in blue. The HLA-DM binding site is shown in yellow (globular domain contacts not shown). The residues that form the epitope for antibodies specific for the empty binding groove are shown in dark blue. The two-headed arrow indicates where the binding-groove width was measured for analysis (distance between centers of mass of residues α60–65 and β65–70). (*B*) The initial decomposition resulting from the choice of MZs is shown schematically. Helices α1 and β1 are shown as two green (A1,A2) and three blue rectangles (B1–B3). Each rectangle represents a helical segment that is linked to adjacent segments by molten zones. Each molten zone can be selectively switched on or off (1/0). (*C*) Example showing test case ${}^{010}T$. The resulting segments are outlined by dotted lines. The six DOFs (three translations and three rotations) for each segment are shown on the top right. To see this figure in color, go online.

Note that we also introduced permanently activated MZs at the end of the helices to allow for the free movement of all the segments (Fig. S1 in the Supporting Material).

Step IV: conformational sampling and evaluation

Once the test cases were defined, we used NMMC (20) to generate the distributions seen in Fig. 2.

Fig. 2 shows the binding-groove width as defined in Fig. 1 *A* and surface area distributions as calculated in Hischenhuber et al. (40) for all eight test cases. For clarity, the test cases are shown in two groups. The first group includes the test cases in which $\Omega_{MZ}^{(2)}$ was activated (Fig. 2, *A* and *B*). The resulting bimodal width and surface area distributions show that the binding groove readily transitions between a wide and a narrow conformation. Depending on the test case, the narrow population is more or less prominent. Test case ${}^{010}T$, for example, exhibits a distribution with clearly defined wide and narrow populations. Note that the distribution was shifted toward the wide population in test cases ${}^{110}T$ and ${}^{111}T$ when $\Omega_{MZ}^{(1)}$, i.e., the α1-1 kink was activated. The second group shows test cases in which $\Omega_{MZ}^{(2)}$ was deactivated (Fig. 2, *C* and *D*). Some narrowing of the binding groove can be observed for test cases ${}^{100}T$ and ${}^{101}T$, but the effect on the surface area is minimal. Generally the binding groove remains in an open conformation when $\Omega_{MZ}^{(2)}$, i.e., the β1-1 kink is kept rigid (${}^{-0-}T$).

Therefore, our customized natural move simulations suggest that the β1-1 kink plays a crucial role in facilitating a conformational change that results in the narrowing of the binding groove.

TABLE 1 All Possible Test Cases that Result from the Initial Decomposition

Test Case	Segments	Number of Segments
${}^{111}T$	$\{S_{A1}, S_{A2}, S_{B1}, S_{B2}, S_{B3}\}$	5
${}^{110}T$	$\{S_{A1}, S_{A2}, S_{B1}, S_{B2+B3}\}$	4
${}^{101}T$	$\{S_{A1}, S_{A2}, S_{B1+B2}, S_{B3}\}$	4
${}^{011}T$	$\{S_{A1+A2}, S_{B1}, S_{B2}, S_{B3}\}$	4
${}^{100}T$	$\{S_{A1}, S_{A2}, S_{B1+B2+B3}\}$	3
${}^{010}T$	$\{S_{A1+A2}, S_{B1}, S_{B2+B3}\}$	3
${}^{001}T$	$\{S_{A1+A2}, S_{B1+B2}, S_{B3}\}$	3
${}^{000}T$	$\{S_{A1+A2}, S_{B1+B2+B3}\}$	2

The set of segments is shown for each test case.

Biological discussion

All MHC class II structures with bound peptide that have been solved to date are structurally highly similar. In the absence of peptide, the MHC II is thought to undergo conformational changes (53,54). However, presumably due to its floppy nature in the absence of peptide (58), the structure of the empty MHC II has not yet been solved by x-ray crystallography. Other experimental techniques have been employed to show that the empty MHC II assumes at least two distinct forms: a peptide-receptive and a peptide-averse form (29,30,50–52). The receptive form mainly exists immediately after peptide dissociation and turns into the averse form within minutes. Given enough time, however, the averse form can isomerize back to the receptive form (50,51).

The structural mechanisms underlying the conversion from receptive to averse are little understood. One simulation study suggested that partial unfolding of the α1 helix gives rise to a helical segment that binds the P1 pocket of the groove in a peptidelike fashion (56). However, this effect

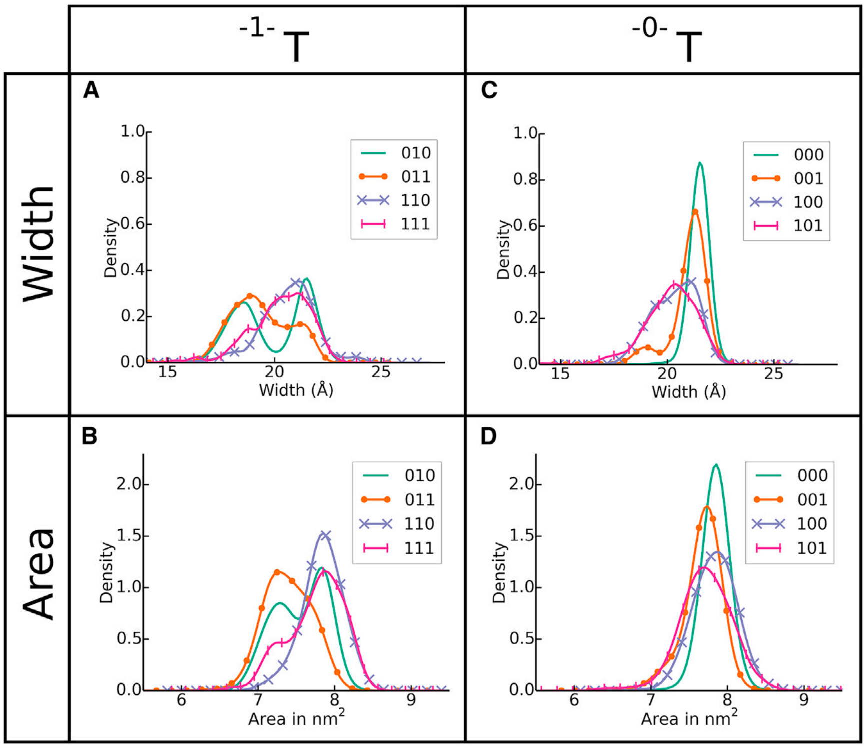

FIGURE 2 Distributions of the binding-groove width and surface area generated during simulation. (*A* and *B*) The left column shows test cases in which molten zone $\Omega_{MZ}^{(2)}$ was activated (^{-1-}T). Note the bimodal width and area distributions, which show that the MHC II binding groove takes on a wide and a narrow binding-groove conformation during simulation. (*C* and *D*) The right column shows test cases where the molten zone $\Omega_{MZ}^{(2)}$ in the β1-1 kink was deactivated (^{-0-}T). Note that the binding-groove area remains stable for these test cases. To see this figure in color, go online.

was abrogated when the protonation state of the starting structure was adjusted (49,57). These studies suggested an involvement of the β1 rather than the α1 helix in the narrowing of the binding groove. In particular, they have shown that the region around the β1-1 kink is highly dynamic (49,57). Interestingly, the β1-1 kink is part of an epitope for two monoclonal antibodies that selectively bind the empty and not the peptide-loaded MHC II (53,55). Additionally, MD simulations on an empty MHC I complex have suggested that the helix, which is the equivalent of the β1-helix in MHC II, is responsible for the closing and opening of the binding groove (59).

In our simulations, we have observed a similar role of the β1 helix in binding-groove plasticity. Only in test cases where $\Omega_{MZ}^{(2)}$ (the β1-1 kink) was active, was a significant narrowing of the binding groove seen (Fig. 2, *A* and *B*). Previous observations in the literature regarding conformational heterogeneity of residues β53–68 around the β1-1 kink have been made (49,53,55–57), which are concordant with our own results suggesting that flexibility in the β1 helix provided by the β1-1 kink leads to a collapse of the binding groove.

Case 2: structural effects of 5-hmC on the DDD

Here we investigated the effect of 5hmC on local basepair arrangement in the DDD: a simple model system that has recently attracted interest due to a new crystal structure with added hydroxymethyl epigenetic marks on cytosines A9 and B9 (33). Two hydroxymethyl configurations were found in this structure. One points toward the backbone phosphate oxygen 5hmC/OP2; the other forms a weak hydrogen bond with the 3′-adjacent G/O6. For the purpose of this case study, we focused on the latter, as it was estimated to be the most prevalent configuration in the crystal (33). A schematic of the system is shown in Fig. 3 *A*. Next, we apply the four main steps of our protocol to investigate the effects of 5hmC on this structure.

Step I: define a hypothesis

Lercher et al. (33) observed that the 5hmC hydroxyl formed a noncanonical hydrogen bond with the 3′-G/O6. This oxygen is already part of a canonical (Watson-Crick) hydrogen bond with the C on the opposing strand. No structural differences between the DDD with and without the epigenetic mark were observed, suggesting that any effects that 5hmC might have on the surrounding basepairs cannot be seen in a static structure. We investigate the hypothesis that the hydroxyl-group on 5hmC subtly interferes with the 3′-adjacent G-C basepair.

Step II: translate hypothesis into natural moves

Given our hypothesis, we defined two sets of cNMs. The first set contained the two torsion angles around bonds C5-C5M ($\Omega_{\phi}^{(1)}$) and C5M-O5 ($\Omega_{\phi}^{(2)}$) in the 5hm epigenetic mark. This gave us control over the orientation of the hydroxyl group during simulation. The second set of cNMs described the collective movement of 5hmC and the 3′-adjacent G, when the MZ between them ($^{*}\Omega_{MZ}^{(1)}$) was

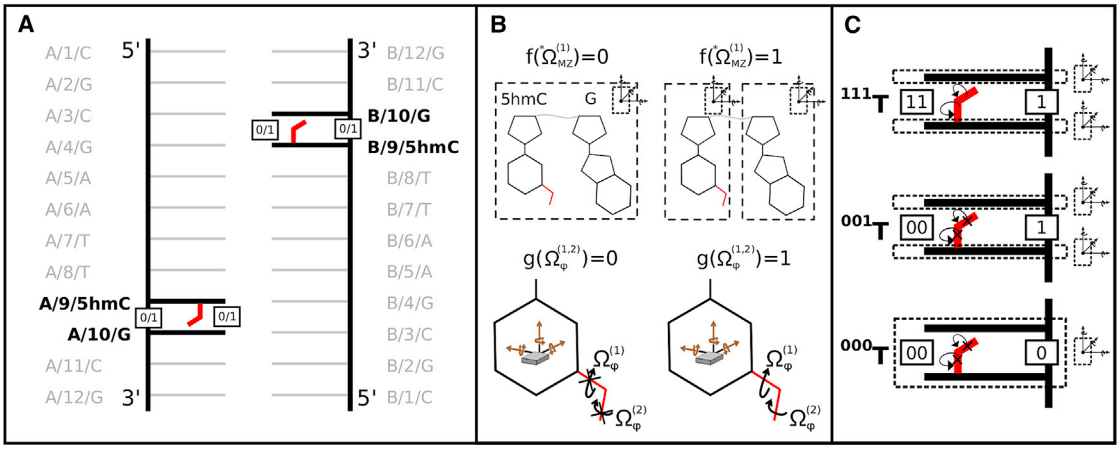

FIGURE 3 Defining cNMs for 5-hydroxymethylcytosine in the DDD. (*A*) Schematic showing the DDD with two added 5-hydroxymethyl (5hm) epigenetic marks. The red lines represent the 5hm epigenetic marks and the thick black horizontal lines represent the bases that are directly affected by the cNMs. (*B*) The gray line connecting the two nucleotides represents an abstracted backbone chain that may undergo chain breaks during NMMC moves. The dotted rectangles show the collective motion of two neighboring nucleotides when the interjacent molten zone $^*\Omega_{MZ}$ is deactivated or activated. The red lines show the epigenetic mark with the arrows highlighting the torsion angles around C5-C5M and C5M-O, the sampling of which may be deactivated or activated, depending on the test case. (*C*) Test cases ^{111}T, ^{001}T, and ^{000}T are shown. The dotted lines show individual or collective DOFs depending on the state of the interjacent MZ (active/inactive). The arrows on the epigenetic marks represent rotations around the two torsion angles of 5hm that may be active or inactive. Note that only one of the two epigenetic marks is shown. However, both modifications are treated equivalently in each case. To see this figure in color, go online.

deactivated. This customized natural move was meant to simulate the stabilizing effect caused by a noncanonical intrastrand hydrogen bond between the two neighboring nucleotides. Fig. 3 *B* shows the cNMs. Note that the depiction of molten zone $^*\Omega_{MZ}^{(1)}$ is an abstraction, as some of the detail was omitted for simplicity. The effect of the cNMs on the distribution of hydrogen-bond distances between the 5hmC hydroxyl and the 3′-G/O6 oxygen is shown in Fig. S2.

Step III: generate test cases

Given the cNMs that we defined above, we get a decomposition vector $\mathbb{D}$ of length 3 (see Materials and Methods). The first two elements refer to rotational freedom along the two torsion angles $\Omega_\phi^{(1)}$ and $\Omega_\phi^{(2)}$ in the hydroxyl group of 5hmC, and the third refers to $^*\Omega_{MZ}^{(1)}$ that consists of the backbone atoms between 5hmC and the 3′-adjacent G. Similar to the protein example, each element in $\mathbb{D}$ can either be on or off (1/0), i.e., the relative arrangement of G and 5hmC in the case of $^*\Omega_{MZ}^{(1)}$ and the sampling of torsion angles included in $\Omega_\phi^{(1)}$ and $\Omega_\phi^{(2)}$ can either be activated or deactivated. Thus, for a decomposition vector $\mathbb{D}_{DNA}: \{g(\Omega_\phi^{(1)}), g(\Omega_\phi^{(2)}), f(^*\Omega_{MZ}^{(1)})\}$ of length 3, we get the following $2^3 = 8$ possible test cases: ^{000}T, ^{001}T, ^{010}T, ^{100}T, ^{011}T, ^{101}T, ^{110}T, and ^{111}T.

Note that we only considered test cases where both of the torsion angles were either active or inactive, as we were only interested in a fully flexible or fixed epigenetic mark for this study. Therefore, we omitted test cases ^{010}T, ^{100}T, ^{011}T, and ^{101}T. The remaining test cases included ^{000}T, ^{001}T, ^{110}T, and ^{111}T. Test case ^{110}T was also ignored as it is very similar to test case ^{000}T due to the deactivated molten zone restraining the orientation of the two neighboring bases. Thus, the set of test cases we included in our study were ^{000}T,^{001}T, and ^{111}T.

Step IV: conformational sampling and evaluation

We ran four sets of simulations of the DDD: the three test cases ^{111}T, ^{001}T, ^{000}T (Fig. 4 *A*), and a simulation without the epigenetic mark that served as a control. Fig. 4 *B* shows the distributions of parameters shear, stretch, and propeller, which changed progressively as we applied the different test cases. Note that we only show the distributions for the basepairs around one of the epigenetic marks, but the effect was seen on both ends. Interestingly, the shear was most affected in the GC basepair 3′-adjacent to x, while the stretch and propeller were mostly changed in the 5′-adjacent basepair. No large differences between the modified ^{111}T system and the unmodified control were observed. However, once the orientation of epigenetic mark was fixed (test case ^{001}T), a subtle shift in the distribution was detected. The effect was further increased when the relative movement between 5hmC and the 3′-adjacent G was deactivated (test case ^{000}T). Changes were also observed in the basepair parameters stagger, buckle, and opening, but the effects were less systematic and did not correspond to the increasing epigenetic signal encoded in our test cases (all basepair parameter values are shown in Fig. S3). We did not investigate changes in the base stack parameters (Fig. S4), as we expected that the noncanonical epigenetic (intrastrand) hydrogen bond formation, which we enforce by customized natural moves, could directly impose particular base stacking. However, we were more interested to study distributions over DNA basepair parameters, which were less directly affected by hydrogen-bond formation between adjacent (on the same strand) bases.

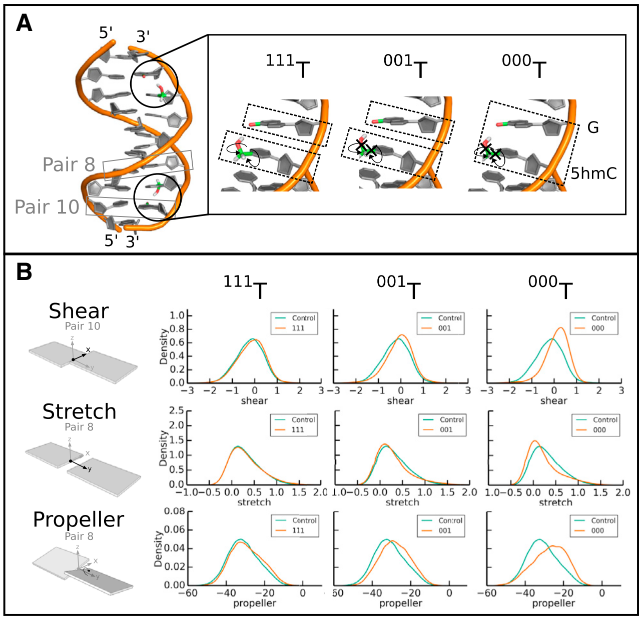

FIGURE 4 the effect of 5-hydroxymethylcytosine on the DDD is amplified by cNMs. (*A*) The DDD is depicted with the backbone in orange and bases in gray. The two 5hmC modifications are colored based on atom type (O, *red*; C, *green*; and H, *white*). The sets of DOFs chosen for the 5hmC modification are shown on the right. Curved arrows indicate free torsional sampling, while the black crosses indicate fixed χ-torsion on rotations around corresponding bonds (χ1, C5-C5M; χ2, C5M-OH). ^{111}T: Full sampling of all torsion angles; ^{001}T: Fixed torsion angles in 5hmC; ^{000}T: Fixed torsion angles in 5hmC and relative orientation between 5hmC and G. (*B*) Distributions of the shear, stretch, and propeller are shown for the three different test cases. Each column compares simulations without modification (control) in green against test cases ^{111}T, ^{001}T, and ^{000}T in orange. The shear is shown for basepair 10 and the stretch and propeller are shown for basepair 8. To see this figure in color, go online.

Biological discussion

A sequence of enzymatic reactions drives a cycle of epigenetic cytosine modifications including 5mC, 5hmC, 5-formylcytosine, and 5-carboxylcytosine (60,61). 5mC has been shown to increase dsDNA stability, which is consistent with its role in gene expression at CpG islands (31). 5hmC, sometimes referred to as the sixth base of the mammalian genome, can partly reverse the 5mC stabilizing given the right sequence context (32) and a study investigating a 27-bp oligonucleotide has observed that 5hmC increases DNA flexibility in MD simulations (62). Several DNA structures with 5hmC epigenetic marks have been solved to date, but no significant structural effects on the DNA helical parameters have been found (33,63,64). This is in contrast to a structure of a DNA dodecamer comprising three 5-formyl CpG sites that showed how 5-formylcytosine causes large structural changes that lead to helical underwinding (65).

To demonstrate how cNMs can be used to study the effects of epigenetic marks, we chose a recent high-resolution structure of the DDD comprising a 5hmC epigenetic modification. When performing traditional natural-move Monte Carlo, we found that the presence of a single 5hmC epigenetic mark in DDD causes only minimal change in some of the helical parameters of the 3′-adjacent basepair. These results agree with the general view that a single 5hmC epigenetic mark has a limited structural effect on the surrounding helical parameters, which makes it difficult to identify experimentally (33,63,64). The results are also in concurrence with Lercher et al. (33), who found that their crystal structures with and without 5hmC were nearly identical with a root-mean-square deviation of 0.35 Å and a 0.8 Å widening of the major groove at the site of modification.

However, using cNMs constrained the 5hmC hydroxyl group in the experimentally determined configuration and thereby increased noncanonical intrastrand hydrogen bonding during simulation and we were able to amplify some of the changes caused by the presence of 5hm. The effect was further increased when we deactivated the relative movement between 5hmC and the 3′-G, thereby effectively emulating the stabilizing effect of an intrastrand hydrogen bond. Thus, using cNMs, we were able to detect and amplify subtle structural effects on DNA helical parameters caused by a single epigenetic mark in the DDD.

DISCUSSION

In this article, we describe a protocol for the testing of hypotheses regarding the functional motions in biological systems. It is based on the natural-move Monte Carlo method that allows for the sampling of conformations given a structural decomposition defined by the researcher.

The use of both cNMMC and NMMC assume the decomposition of the molecular system into segments and molten zones. The implementation methodology (19,20) of NMMC follows a segment-centric approach; if adjacent segments move with respect to each other, their translational and orientational updates are independent; otherwise, a larger segment including the adjacent segments is defined.

In cNMMC, end-users may consider each molten zone as active/inactive or 1/0 so that adjacent segments may move independently or synchronously. Using this MZ-centric approach, each set of DOFs (each test case) is associated with a binary string so that test cases can be easily organized and annotated in a systematic and high-throughput manner. In this way, cNMMC reduces the technical barrier to the use of the NMMC approach (19,20,23,28) to study the anatomy of necessary and sufficient sets of DOFs responsible for molecular function.

The efficient chain closure algorithm (19) allows the user to introduce arbitrary DOFs into a system without substantially compromising computational run-time.

We used this customization capability as the basis for a protocol for the investigation of structural mechanisms. The protocol allows for an investigative strategy using a range of simulations with distinct sets of customized natural

moves to test hypotheses concerning the functional motions in biological systems.

In molecular biology, a classical approach to testing hypotheses regarding the function of a certain gene is to interfere with its expression and see what happens to the organism. Similarly, in experimental structural biology, residues can be mutated or removed to identify functional regions such as protein-binding or enzyme-active sites (66).

However, to our knowledge, this concept of reverse engineering has not been used for investigation of functional motions in simulations. For the first time, to our knowledge, our cNMs protocol enables the testing of hypotheses regarding the functional motions of a biological system by allowing the user to enhance or restrict the movement of certain structural regions.

Hypotheses may be derived from biological intuition or computational and/or experimental methods such as ED-CG (15,67), principal component analysis (PCA) (68), elastic network models (69), normal mode analysis (NMA) (18), and nuclear magnetic resonance (NMR) (70).

ED-CG provides information on essential motion by PCA of MD simulations (15) or an elastic network model of a single atomic structure (67). Similarly, low-frequency modes calculated by NMA are often used to approximate collective functional motions in biomolecules (18). In most cases, all the important modes are contained in the normal mode basis set. Thus, NMA provides valuable information on the collective motion of biological systems that may guide the design of natural moves. However, it is often unclear which modes are functionally relevant, as the normal mode basis set contains a range of possible candidates (71). cNMMC simulations may be used to identify functionally relevant modes with different sets of natural moves that represent unique low-frequency modes.

Furthermore, NMA or PCA can be costly, as the computational complexity associated with these methods is $O(N^3)$ (worst case), where N is the number of atoms in the system. This is due to solving the underlying eigenvalue problem associated with the Hessian matrix. Advanced solvers might produce better scaling, e.g., $O(N^3)$ with $c < 3$; but even by using these advanced methods, the computational cost associated with NMA or PCA will dominate $O(N)$, which is the time complexity of modern algorithms (72,73) for calculating most statistical (22) or empirical (42) force fields. On the contrary, the worst-case time complexity of NMMC is strictly $O(N)$, because we developed a chain closure algorithm (22) that has linear complexity, $O(N_d)$, in terms of the number of DOFs; here, N_d was used to solve the chain closure (the inverse kinematic) problem. Because $N_d < N$, the application of natural moves, unlike the calculation of NMA or PCA, will never dominate the computational cost of molecular simulations. This is the main quantifiable advantage of using cNMMC instead of NMA or PCA.

A less quantifiable but still notable advantage of cNMMC compared to NMA (or PCA) is that it allows the use of highly unconventional experimentally inferred DOFs such as the hand-shaking motion of adjacent subunits in a chaperonin (23). These experimentally derived moves are not necessarily associated with or dependent on a single conformation (NMA) or conformational ensembles (PCA). They can be simply defined without any limitation to test any experimental observation or intuition. Therefore, NMMC not just supports moves derived using PCA or NMA, but any type of moves (e.g., move any part of the system and the rest will deform to follow the change).

The scope of NMMC also differs from the scope of NMA or PCA. NMA (PCA) takes a minimum energy conformation (conformational ensemble) as its input and outputs collective motions or deformations of the molecular system. On the contrary, NMMC takes any collective motion (including but not limited to the ones derived from PCA or NMA) as input and provides distributions as output by exploring the relevant conformational space orders of magnitude more efficiently (22) than conventional methods such as Cartesian Monte Carlo or MD. In this capacity, NMMC has linear $O(N)$ scaling, so it is perfectly fitted to high-throughput testing of customized natural moves. NMA-based Monte Carlo would require the successive recalculation of normal modes in concert with the changing molecular conformation and the computational cost would scale as $O(N^3)$ (worst case).

Thus, cNMMC should be considered as a complementary approach to NMA or PCA. For example, NMA or PCA can be used in the construction of natural moves and cost-efficient NMMC can explore the conformational space. cNMMC can also be used to test the validity of low-frequency normal mode-based natural moves while exploring the conformational space distant from the minimum energy conformation used to generate the normal mode.

Similarly to the above discussion on how cNMMC differs from NMA or PCA, we would like to highlight the differences of cNMMC over MD or Monte Carlo methods with imposed constraints. The latter two methods enable the user to impose constraints on certain DOFs. In contrast, the use of cNMMC primarily facilitates conformational change along a set of user-defined or experimentally inferred DOFs (referred to as natural moves); other DOFs are treated as subordinate (but not constrained) to fully facilitate the exploration of the conformational space along natural moves. This is a very different strategy from Cartesian (or generalized) coordinate-based exploration of the conformational space with constraints, regardless of whether the exploration algorithm is MD- or Monte Carlo-based. Due to the benefits of natural moves, where chain breakage is followed by closure, any part of the molecular assembly can be moved and the necessary subordinate or dependent DOFs will be rearranged to maintain the integrity of the system. This strategy provides users with the opportunity to focus on the essential moves or molecular deformations rather than the less important DOFs.

By the straightforward definition of customized natural moves, cNMMC can facilitate the robust compilation of experimentally inferred (23) molecular motion into a molecular simulation protocol. This advantage is particularly relevant for computational structural biology, given the complexity and diversity of biomolecular architectures. Focusing on the natural moves as opposed to the corresponding constraints can provide a more intuitive way to describe, classify, and ultimately understand the mechanisms underpinning functionally relevant motions. The characterization of dynamics in biological molecules is one of the grand challenges of computational structural biology and biophysics, and can only be tackled by the tight collaboration of computational and experimental scientists. To tighten this partnership, the use of cNMMC can catalyze more active engagement of experimental biophysical scientists, who often have extensive experience working on a given biological system in conducting these types of molecular simulations.

The more quantifiable advantage of cNMMC compared to MDs (or Monte Carlo) with imposed constraints is the large speed gain from reducing the number of essential DOFs. In a cNMMC protocol the investigation is commonly restricted to a few DOFs (e.g., 6+; orientational and translational parameters of a structural segment plus a few internal dihedral and bond angle DOFs), whereas it is less intuitive for a general user, who might not be specialized in molecular simulations, to automate the procedure for imposing constraints on the remaining DOFs. In addition, the use of dependent DOFs (see the Supporting Material), which significantly facilitates exploration along desired motions, is another unique feature of the presented technology compared to constrained MDs or Monte Carlo methods.

With the advantage of being able to define moves liberally and sample conformations along these moves very efficiently, we managed to address applications (20,21,23,25,44) that were not feasible before NMMC. For example, the latest application (21) demonstrated that we could speed up simulations by orders of magnitude compared to MDs, while still being able to reproduce experimental observables. With cNMMC, these computational experiments will become more accessible to a wider scientific community including experimental laboratories.

As described above with NMA and PCA, cNMMC is best used as a complementary method to constrained MDs (or Monte Carlo), which could refine our understanding of systems with DOFs that cNMMC predicts to be relevant.

As well as other computational methods, experimental information on collective motions derived from NMR data (70) may also be used to guide the design of cNMs. A range of methods already exist that use NMR data to complement MD simulations (4).

Additionally, preexisting expert knowledge is central to generating new ideas. The cNMMC protocol presented here is a first step to bridging the gap between the biological intuition of scientists and molecular simulations by allowing the introduction of arbitrary DOFs for the investigation of conformational changes and mechanisms.

In summary, we describe a strategy for the systematic use of customized natural moves to test hypotheses regarding functional motions and have demonstrated the protocol's ability to provide biological insight into a protein and a DNA system.

CONCLUSION

We demonstrated the use of a computationally cheap protocol that uses customized natural moves to investigate the nature of structural changes in a protein and an epigenetically modified DNA system. For each system we generated hypotheses derived from observations in the literature as well as our own preliminary simulation results and performed simulations on a set of different customized natural moves. We showed that this enables the systematic testing of DOFs, which allows for the investigation of causal relationships regarding functional motions in biological systems.

SUPPORTING MATERIAL

Supporting Materials and Methods and four figures are available at http://www.biophysj.org/biophysj/supplemental/S0006-3495(16)30474-X.

AUTHOR CONTRIBUTIONS

S.D., B.K., C.M.D., and P.M. conceived and designed the experiments; S.D. performed the experiments; S.D., C.M.D., and P.M. analyzed the data; B.K. and P.M. contributed reagents/materials/analysis tools; S.D. and P.M. wrote the article; and B.K. and C.M.D. revised the article critically.

ACKNOWLEDGMENTS

We acknowledge the use of the 1st Intel Xeon Phi Access Programme in the Hartree Centre of the UK Science & Technology Facilities Council and the use of the Advanced Research Computing facility at the University of Oxford in carrying out this work. This work was supported by the Engineering and Physical Sciences Research Council [EP/G03706X/1].

SUPPORTING CITATIONS

Reference (74) appears in the Supporting Material.

REFERENCES

1. Henzler-Wildman, K., and D. Kern. 2007. Dynamic personalities of proteins. *Nature.* 450:964–972.
2. Moraitakis, G., A. G. Purkiss, and J. M. Goodfellow. 2003. Simulated dynamics and biological macromolecules. *Rep. Prog. Phys.* 66:383–406.
3. van Gunsteren, W. F., J. Dolenc, and A. E. Mark. 2008. Molecular simulation as an aid to experimentalists. *Curr. Opin. Struct. Biol.* 18:149–153.
4. Esteban-Martín, S., R. Bryn Fenwick, and X. Salvatella. 2012. Synergistic use of NMR and MD simulations to study the structural

heterogeneity of proteins. *Wiley Interdiscip. Rev. Comput. Mol. Sci.* 2:466–478.

5. Karplus, M., and R. Lavery. 2014. Significance of molecular dynamics simulations for life sciences. *Isr. J. Chem.* 54:1042–1051.
6. McCammon, J. A., B. R. Gelin, and M. Karplus. 1977. Dynamics of folded proteins. *Nature.* 267:585–590.
7. Dror, R. O., R. M. Dirks, …, D. E. Shaw. 2012. Biomolecular simulation: a computational microscope for molecular biology. *Annu. Rev. Biophys.* 41:429–452.
8. Freddolino, P. L., A. S. Arkhipov, …, K. Schulten. 2006. Molecular dynamics simulations of the complete satellite tobacco mosaic virus. *Structure.* 14:437–449.
9. Beberg, A. L., D. L. Ensign, …, V. S. Pande. 2009. Folding@Home: lessons from eight years of volunteer distributed computing. *In* 2009 IEEE International Symposium on Parallel & Distributed Processing. Institute of Electrical and Electronics Engineers, Piscataway, NJ. 1–8.
10. Lindorff-Larsen, K., S. Piana, …, D. E. Shaw. 2011. How fast-folding proteins fold. *Science.* 334:517–520.
11. Orozco, M. 2014. A theoretical view of protein dynamics. *Chem. Soc. Rev.* 43:5051–5066.
12. Bernardi, R. C., M. C. R. Melo, and K. Schulten. 2015. Enhanced sampling techniques in molecular dynamics simulations of biological systems. *Biochim. Biophys. Acta.* 1850:872–877.
13. Duan, M., J. Fan, …, S. Huo. 2013. Evaluation of dimensionality-reduction methods from peptide folding-unfolding simulations. *J. Chem. Theory Comput.* 9:2490–2497.
14. Saunders, M. G., and G. A. Voth. 2013. Coarse-graining methods for computational biology. *Annu. Rev. Biophys.* 42:73–93.
15. Zhang, Z., L. Lu, …, G. A. Voth. 2008. A systematic methodology for defining coarse-grained sites in large biomolecules. *Biophys. J.* 95:5073–5083.
16. Bahar, I., T. R. Lezon, …, E. Eyal. 2010. Global dynamics of proteins: bridging between structure and function. *Annu. Rev. Biophys.* 39: 23–42.
17. Perilla, J. R., and T. B. Woolf. 2012. Towards the prediction of order parameters from molecular dynamics simulations in proteins. *J. Chem. Phys.* 136:164101.
18. Bahar, I., and A. J. Rader. 2005. Coarse-grained normal mode analysis in structural biology. *Curr. Opin. Struct. Biol.* 15:586–592.
19. Minary, P., and M. Levitt. 2010. Conformational optimization with natural degrees of freedom: a novel stochastic chain closure algorithm. *J. Comput. Biol.* 17:993–1010.
20. Sim, A. Y. L., M. Levitt, and P. Minary. 2012. Modeling and design by hierarchical natural moves. *Proc. Natl. Acad. Sci. USA.* 109:2890–2895.
21. Knapp, B., S. Demharter, …, P. Minary. 2016. Exploring peptide/MHC detachment processes using hierarchical natural move Monte Carlo. *Bioinformatics.* 32:181–186.
22. Minary, P., and M. Levitt. 2008. Probing protein fold space with a simplified model. *J. Mol. Biol.* 375:920–933.
23. Zhang, J., P. Minary, and M. Levitt. 2012. Multiscale natural moves refine macromolecules using single-particle electron microscopy projection images. *Proc. Natl. Acad. Sci. USA.* 109:9845–9850.
24. Sim, A. Y. L., P. Minary, and M. Levitt. 2012. Modeling nucleic acids. *Curr. Opin. Struct. Biol.* 22:273–278.
25. Moraga, I., G. Wernig, …, K. C. Garcia. 2015. Tuning cytokine receptor signaling by re-orienting dimer geometry with surrogate ligands. *Cell.* 160:1196–1208.
26. Cresswell, P. 1994. Assembly, transport, and function of MHC class II molecules. *Annu. Rev. Immunol.* 12:259–293.
27. Jones, E. Y., L. Fugger, …, C. Siebold. 2006. MHC class II proteins and disease: a structural perspective. *Nat. Rev. Immunol.* 6:271–282.
28. Knapp, B., S. Demharter, …, C. M. Deane. 2015. Current status and future challenges in T-cell receptor/peptide/MHC molecular dynamics simulations. *Brief. Bioinform.* 16:1035–1044.
29. Sadegh-Nasseri, S., and H. M. McConnell. 1989. A kinetic intermediate in the reaction of an antigenic peptide and I-Ek. *Nature.* 337:274–276.
30. Natarajan, S. K., M. Assadi, and S. Sadegh-Nasseri. 1999. Stable peptide binding to MHC class II molecule is rapid and is determined by a receptive conformation shaped by prior association with low affinity peptides. *J. Immunol.* 162:4030–4036.
31. Münzel, M., D. Globisch, and T. Carell. 2011. 5-Hydroxymethylcytosine, the sixth base of the genome. *Angew. Chem. Int. Ed. Engl.* 50:6460–6468.
32. Thalhammer, A., A. S. Hansen, …, C. J. Schofield. 2011. Hydroxylation of methylated CpG dinucleotides reverses stabilisation of DNA duplexes by cytosine 5-methylation. *Chem. Comm.* 47:5325–5327.
33. Lercher, L., M. A. McDonough, …, C. J. Schofield. 2014. Structural insights into how 5-hydroxymethylation influences transcription factor binding. *Chem. Comm.* 50:1794–1796.
34. Knapp, B., J. Dunbar, and C. M. Deane. 2014. Large scale characterization of the LC13 TCR and HLA-B8 structural landscape in reaction to 172 altered peptide ligands: a molecular dynamics simulation study. *PLoS One*. 10:e1003748.
35. Minary, P. 2007. Methodologies for Optimization and Sampling in Computational Studies (MOSAICS), Vers 3.9. Department of Computer Science, University of Oxford, Oxford, UK. *http://www.cs.ox.ac.uk/mosaics*.
36. Hunter, J. D. 2007. Matplotlib: A 2D graphics environment. *Comput. Sci. Eng.* 9:90–95.
37. McKinney, W. 2011. pandas: a Foundational Python Library for Data Analysis and Statistics. Presented at PyHPC2011, November 18, 2011. http://pandas.pydata.org/talks.html.
38. Pos, W., D. K. Sethi, …, K. W. Wucherpfennig. 2012. Crystal structure of the HLA-DM-HLA-DR1 complex defines mechanisms for rapid peptide selection. *Cell.* 151:1557–1568.
39. Michaud-Agrawal, N., E. J. Denning, …, O. Beckstein. 2011. MDAnalysis: a toolkit for the analysis of molecular dynamics simulations. *J. Comput. Chem.* 32:2319–2327.
40. Hischenhuber, B., H. Havlicek, …, B. Knapp. 2013. Differential geometric analysis of alterations in MH α-helices. *J. Comput. Chem.* 34:1862–1879.
41. DeLano, W. L. 2014. The PyMOL Molecular Graphics System, Ver. 1.7.4. Schrödinger LLC. *http://www.schrodinger.com/*.
42. Pérez, A., I. Marchán, …, M. Orozco. 2007. Refinement of the AMBER force field for nucleic acids: improving the description of α/γ conformers. *Biophys. J.* 92:3817–3829.
43. Rohs, R., C. Etchebest, and R. Lavery. 1999. Unraveling proteins: a molecular mechanics study. *Biophys. J.* 76:2760–2768.
44. Minary, P., and M. Levitt. 2014. Training-free atomistic prediction of nucleosome occupancy. *Proc. Natl. Acad. Sci. USA.* 111:6293–6298.
45. Lu, X. J., and W. K. Olson. 2003. 3DNA: a software package for the analysis, rebuilding and visualization of three-dimensional nucleic acid structures. *Nucleic Acids Res.* 31:5108–5121.
46. van de Rijn, M., C. Bernabeu, …, C. Terhorst. 1984. Recognition of HLA-A2 by cytotoxic T lymphocytes after DNA transfer into human and murine cells. *Science.* 226:1083–1085.
47. Schreier, M. H., N. N. Iscove, …, H. von Boehmer. 1980. Clones of killer and helper T cells: growth requirements, specificity and retention of function in long-term culture. *Immunol. Rev.* 51:315–336.
48. Vyas, J. M., A. G. van der Veen, and H. L. Ploegh. 2008. The known unknowns of antigen processing and presentation. *Nat. Rev. Immunol.* 8:607–618.
49. Yaneva, R., S. Springer, and M. Zacharias. 2009. Flexibility of the MHC class II peptide binding cleft in the bound, partially filled, and empty states: a molecular dynamics simulation study. *Biopolymers.* 91:14–27.

50. Rabinowitz, J. D., M. Vrljic, …, H. M. McConnell. 1998. Formation of a highly peptide-receptive state of class II MHC. *Immunity.* 9:699–709.
51. Joshi, R. V., J. A. Zarutskie, and L. J. Stern. 2000. A three-step kinetic mechanism for peptide binding to MHC class II proteins. *Biochemistry.* 39:3751–3762.
52. Kasson, P. M., J. D. Rabinowitz, …, H. M. McConnell. 2000. Kinetics of peptide binding to the class II MHC protein I-Ek. *Biochemistry.* 39:1048–1058.
53. Zarutskie, J. A., A. K. Sato, …, L. J. Stern. 1999. A conformational change in the human major histocompatibility complex protein HLA-DR1 induced by peptide binding. *Biochemistry.* 38:5878–5887.
54. Carven, G. J., and L. J. Stern. 2005. Probing the ligand-induced conformational change in HLA-DR1 by selective chemical modification and mass spectrometric mapping. *Biochemistry.* 44:13625–13637.
55. Carven, G. J., S. Chitta, …, L. J. Stern. 2004. Monoclonal antibodies specific for the empty conformation of HLA-DR1 reveal aspects of the conformational change associated with peptide binding. *J. Biol. Chem.* 279:16561–16570.
56. Painter, C. A., A. Cruz, …, Z. Zavala-Ruiz. 2008. Model for the peptide-free conformation of class II MHC proteins. *PLoS One.* 3:e2403.
57. Rupp, B., S. Günther, …, R. Kühne. 2011. Characterization of structural features controlling the receptiveness of empty class II MHC molecules. *PLoS One.* 6:e18662.
58. Sadegh-Nasseri, S., and R. Germain. 1991. A role for peptide in determining MHC class II structure. (Letter). *Nature.* 353:167–170.
59. Zacharias, M., and S. Springer. 2004. Conformational flexibility of the MHC class I $\alpha1$-$\alpha2$ domain in peptide bound and free states: a molecular dynamics simulation study. *Biophys. J.* 87:2203–2214.
60. Goll, M. G., and T. H. Bestor. 2005. Eukaryotic cytosine methyltransferases. *Annu. Rev. Biochem.* 74:481–514.
61. Ito, S., L. Shen, …, Y. Zhang. 2011. Tet proteins can convert 5-methylcytosine to 5-formylcytosine and 5-carboxylcytosine. *Science.* 333:1300–1303.
62. Wanunu, M., D. Cohen-Karni, …, M. Drndic. 2011. Discrimination of methylcytosine from hydroxymethylcytosine in DNA molecules. *J. Am. Chem. Soc.* 133:486–492.
63. Renciuk, D., O. Blacque, …, B. Spingler. 2013. Crystal structures of B-DNA dodecamer containing the epigenetic modifications 5-hydroxymethylcytosine or 5-methylcytosine. *Nucleic Acids Res.* 41:9891–9900.
64. Szulik, M. W., P. S. Pallan, …, M. P. Stone. 2015. Differential stabilities and sequence-dependent base pair opening dynamics of Watson-Crick base pairs with 5-hydroxymethylcytosine, 5-formylcytosine, or 5-carboxylcytosine. *Biochemistry.* 54:1294–1305.
65. Raiber, E.-A., P. Murat, …, S. Balasubramanian. 2015. 5-Formylcytosine alters the structure of the DNA double helix. *Nat. Struct. Mol. Biol.* 22:44–49.
66. Morrison, K. L., and G. A. Weiss. 2001. Combinatorial alanine-scanning. *Curr. Opin. Chem. Biol.* 5:302–307.
67. Zhang, Z., J. Pfaendtner, …, G. A. Voth. 2009. Defining coarse-grained representations of large biomolecules and biomolecular complexes from elastic network models. *Biophys. J.* 97:2327–2337.
68. Balsera, M. A., W. Wriggers, …, K. Schulten. 1996. Principal component analysis and long time protein dynamics. *J. Phys. Chem.* 100:2567–2572.
69. Atilgan, A. R., S. R. Durell, …, I. Bahar. 2001. Anisotropy of fluctuation dynamics of proteins with an elastic network model. *Biophys. J.* 80:505–515.
70. Ravera, E., L. Salmon, …, C. Luchinat. 2014. Insights into domain-domain motions in proteins and RNA from solution NMR. *Acc. Chem. Res.* 47:3118–3126.
71. Ma, J. 2005. Usefulness and limitations of normal mode analysis in modeling dynamics of biomolecular complexes. *Structure.* 13: 373–380.
72. Mattson, W., and B. M. Rice. 1999. Near-neighbor calculations using a modified cell-linked list method. *Comput. Phys. Commun.* 119: 135–148.
73. Yao, Z., J.-S. Wang, …, M. Cheng. 2004. Improved neighbor list algorithm in molecular simulations using cell decomposition and data sorting method. *Comput. Phys. Commun.* 161:27–35.
74. Metropolis, N., A. W. Rosenbluth, …, E. Teller. 1953. Equation of state calculations by fast computing machines. *J. Chem. Phys.* 21:1087.

Biophysical Journal, Volume *111*

Supplemental Information

Modeling Functional Motions of Biological Systems by Customized Natural Moves

Samuel Demharter, Bernhard Knapp, Charlotte M. Deane, and Peter Minary

Modelling Functional Motions of Biological Systems by Customised Natural Moves - Supporting Material

S Demharter, Dr B Knapp, Prof CM Deane, Dr P Minary

Supporting Text

Natural move Monte Carlo sampling

The algorithm. Natural move Monte Carlo (NMMC) aims to sample the conformational space along user defined independent degrees of freedom X_i, which are described for both current models (for Case 1-2) in (1). Given this initial choice, the method generates canonical distributions along X_i over an effective energy surface $\tilde{E}$, which is defined by equation 1 below. Since the proposal kernel (1) along X_i is symmetrical, we use classical Metropolis Monte Carlo (2), which satisfies detailed balance, to sample the different states of X_i. Numerical experiments (3) demonstrate the accuracy (convergence to limiting distributions) and effectiveness (rate of convergence) of this approach.
In NMMC all degrees of freedom, X are partitioned into independent (X_i) and dependent (X_d) degrees of freedom (DoF). For example, X_i represent the independent orientational, translational or internal motions of structural fragments in a molecular chain, whereas X_d are the DoFs that are instantaneously minimised to facilitate exploration along X_i and preserve the integrity of the molecular chain(s) through chain closure(s). Thus, the effective potential over X_i is defined as

$$\tilde{E}(X_i) = \min_{X_d}\Big\{E(X_i \cup X_d)\Big\}. \quad (1)$$

Therefore, natural move Monte Carlo is analogous to a Metropolis sampling (2) exploring state space spanned by X_i over the energy surface $\tilde{E}$. The most unique feature of NMMC is how the complex moves are generated. This is described below.

Implementation. The basic principle is that each new configuration during a proposal step is obtained via a combined chain breakage closure algorithm. This composite proposal kernel includes a stochastic proposal to update X_i followed by finding the most optimal (with respect to the new X_i) arrangement along X_d. This scheme can accelerate the conformational search for possible arrangements of *a priori* defined structural segments or regions (e.g. groups of segments) and is also free of any limitations caused by the lever-arm effects of distant torsional changes, which leads to increasingly (by chain length) low acceptance rates of dihedral moves. Thus, NMMC can be applied for any system regardless of size.
While the above description is general, the exact definition of independent X_i and dependent X_d degrees of freedom should be custom tailored to the model of interest. For the coarse-grained protein model of Case 1 and for the all-atom DNA model of Case 2 X_i and X_d are described in detail in (1).
Numerical experiments. In MCMC simulations it is generally regarded that an acceptance rate of ~0.4 is optimal when a single parameter (one independent variable of X_i) is updated and ~0.2 when a group of parameters (all independent variables in X_i) are updated. Given that we update X_i based on a multivariate normal distribution (1), we consider acceptance rates for natural moves in interval [0.2, 0.3] optimal and rates [0.15, 0.75] generally acceptable.
For replica exchange, we consider acceptance rates for adjacent temperature replica exchange of ~0.2 as optimal and rates in the interval [0.1, 0.3] as acceptable. The choice for these rates are based on considerations such as the sufficient relaxation time of individual Markov chains and the probability of ‘coast to coast’ visits of individual replicas.

Comparing results from different test cases

Each test case of the protocol is an independent model, in which the available conformational space is a subspace of C_f, the domain that includes all functionally relevant conformational variability. C_f is usually chosen to be a proper subspace of the

'full domain', C; $C_f \subset C$, e.g. C could be spanned by the Cartesian degrees of freedom (DoFs) and C_f by dihedral angles about single bonds and bond angles between bonds that an atom forms.
Each test case features some restricted set of DoFs spanning the state space $C_i \subseteq C_f \subset C$ for all $i = 1, , N_T$, where N_T is the number of test cases. Note, that the full domain, C is equipped with an energy function (the original energy surface), $E : C \to \mathbb{R}$ and the energy surface for a given test case is given by the function, E_i, which is a restriction of E to C_i and defined as $E_i : C_i \to \mathbb{R}, E_i(x) = E(x)$, for all $x \in C_i$. Thus, each test case is an independent model featured by C_i and the corresponding energy surface, E_i.
In spite of each test case being associated with its own state space, C_i distributions (over structural observables) obtained for different test cases can be compared to assess the contribution of a particular DoF (e.g. the relative motion of two adjacent helices enabled by a central kink) to functional motions (e.g. changes in MHC-II binding groove area and width). For example, let $\alpha : C \to \mathbb{R}$ be a structural observable and let $P_i(\alpha), i = 1, , N_T$ be the normalised numerical distributions over α we obtain for each test case via performing independent natural move Monte Carlo simulations covering each state space, $C_i, i = 1, , N_T$.
For the protein (Case 1) study we assess some features (e.g. bimodal) of these distributions $P_i(\alpha)$ to identify the DoFs that are essential and ones that are less critical to produce that feature, which may be linked to important biological function. For example, if the binding groove width distribution is bimodal then the MHC binding groove can exhibit two stable conformations (open and closed) even in the absence of the peptide. By systematically grouping all $P_i(\alpha)$ that exhibit this behaviour from those that do not, we can identify the underpinning essential DoFs responsible for this phenomenon. In a similarly qualitative but systematic approach the DNA (Case 2) study compares distributions for test cases to purely identify the existence and directionality of effects a chemical modification imposes on the DNA structural parameters. Our robust initial search can identify test cases or phenomena that could be further investigated by molecular dynamics to obtain refined quantitative information.

Supporting Figures

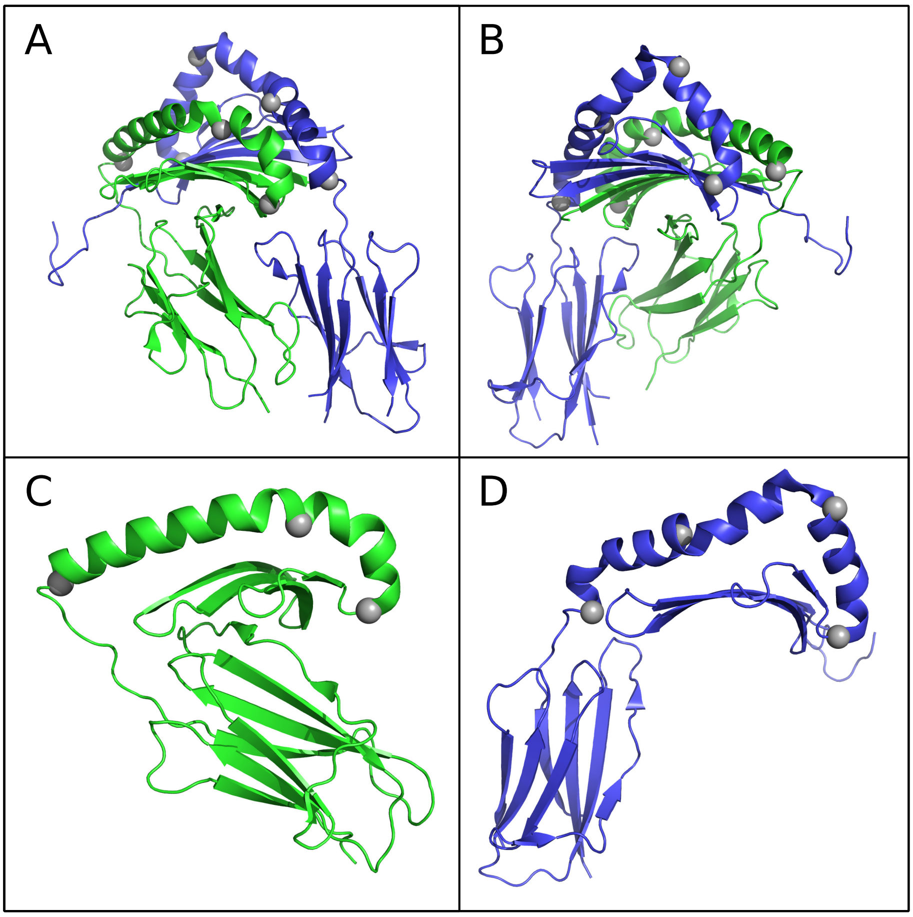

Figure S1: **Molten zones in MHC II. A,B** MHC II is shown in cartoon representation. Chains A and B are coloured in green and blue, respectively. The molten zones are depicted as grey spheres. **C** Chain A and its three molten zones are shown. **D** Chain B and its four molten zones are shown.

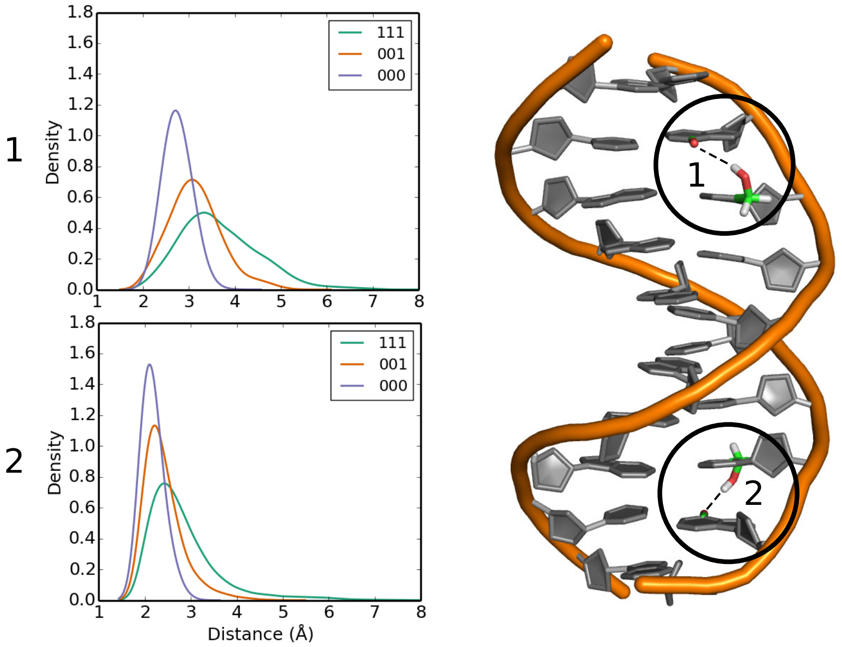

Figure S2: **The effect of customised natural moves on an intra-strand hydrogen bond.** Distance distributions of the two non-canonical hydrogen bonds between the hydroxyl hydrogens on 5hmC and the O6 oxygen of the 3'-adjacent guanine as highlighted on the right. All three test cases are shown. The X-ray structure, which we used as our starting structure, is not totally symmetric so we do not expect totally symmetrical effects as we move from ^{111}T to ^{000}T.

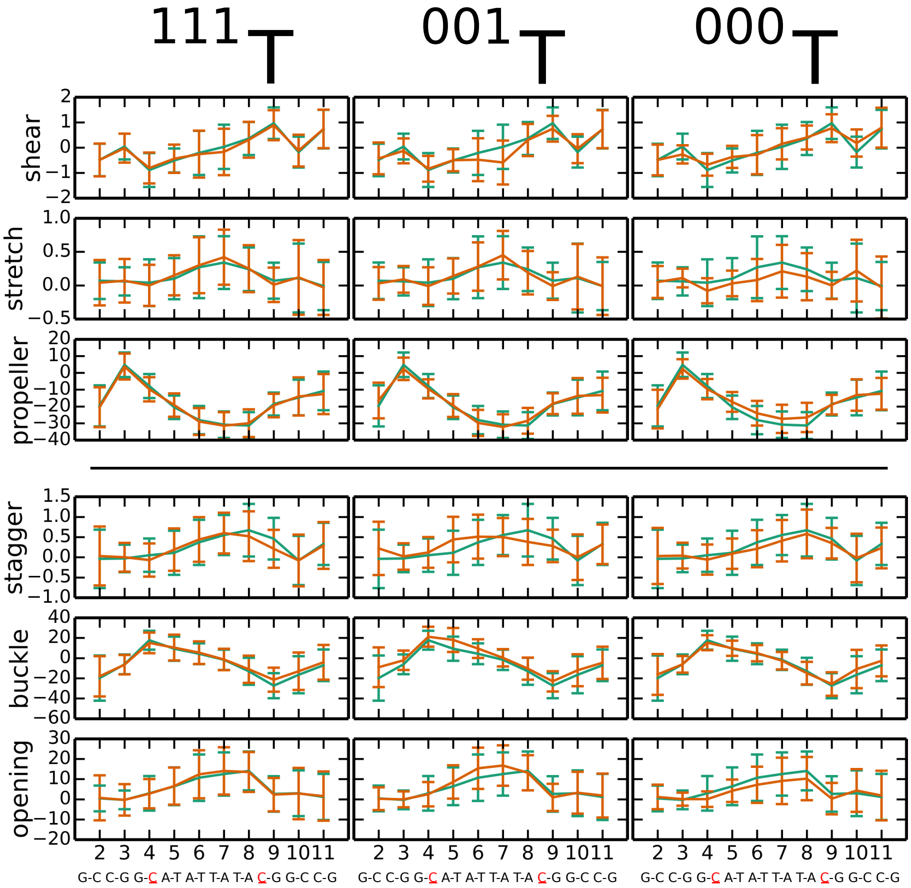

Figure S3: **The effect of three test cases on the base pair parameters.** The parameters for all base pairs in the three test cases ^{111}T, ^{001}T and ^{000}T (orange) are compared against the control simulation without modification (green). The top half of the figure shows the three parameters shown in Fig 3. All other parameters do not show any systematic changes caused by the customised natural moves. Displacement parameters (shear, stretch, stagger) are shown in Ångstrom and angular parameters (buckle, propeller, opening) are shown in degrees. The vertical bars show the standard deviation. The red underlined characters show the positions of the epigenetic mark.

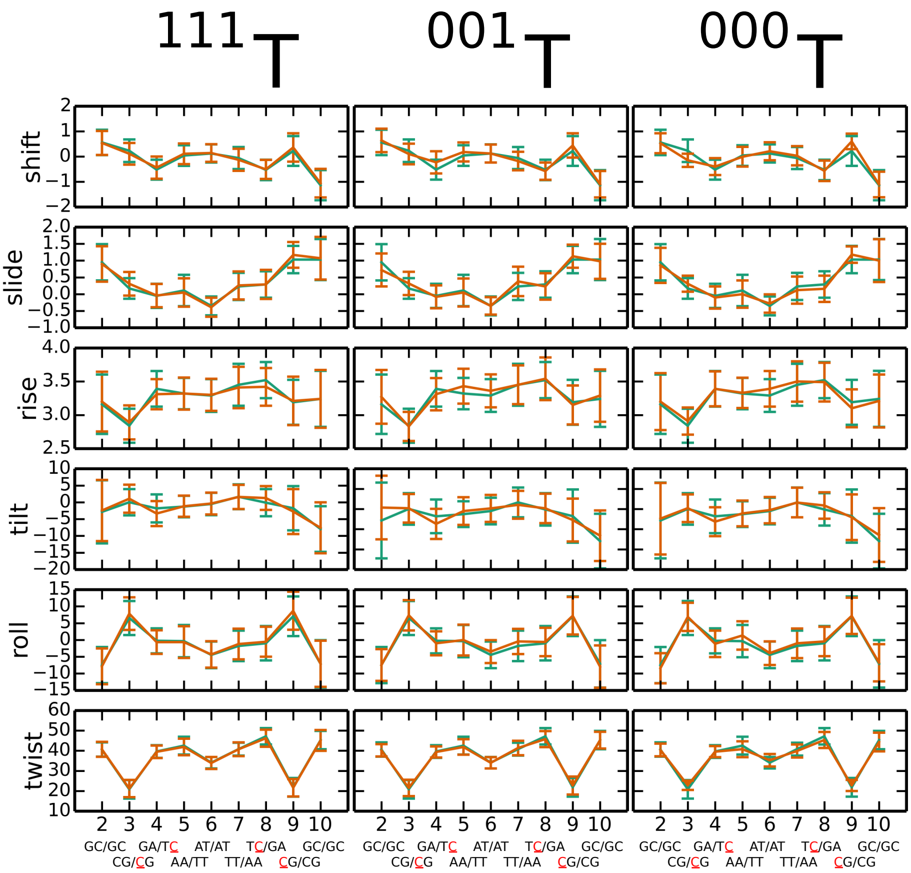

Figure S4: **The effect of three test cases on the base stack parameters.** The parameters for the three test cases ^{111}T, ^{001}T and ^{000}T (orange) are compared against the control simulation without modification (green). No systematic changes were observed in any of the test cases. Displacement parameters (shift, slide, rise) are shown in Ångstrom and angular parameters (tilt, roll, twist) are shown in degrees. The vertical bars show the standard deviation. The red underlined characters show the positions of the epigenetic mark.

Supporting References

1. Minary, P., and M. Levitt, 2010. Conformational optimization with natural degrees of freedom: a novel stochastic chain closure algorithm. J. Comput. Biol. 17:993–1010.
2. Metropolis, N., A. W. Rosenbluth, M. N. Rosenbluth, A. H. Teller, and E. Teller, 1953. Equation of State Calculations by Fast Computing Machines. J. Chem. Phys. 21:1087.
3. Sim, A. Y. L., M. Levitt, and P. Minary, 2012. Modeling and design by hierarchical natural moves. P. Natl. Acad. Sci. 109:2890–5.
